# CFTR—a (novel) target in ARDS: Commentary on “Loss of endothelial CFTR drives barrier failure and edema formation in lung infection and can be targeted by CFTR potentiation”, Erfinanda et al., Sci. Transl. Med. 14, eabg8577 (2022)

**DOI:** 10.1007/s00424-023-02800-4

**Published:** 2023-02-27

**Authors:** Manfred Frick

**Affiliations:** Institute of General Physiology, Ulm University, Ulm, Germany

The *cystic fibrosis transmembrane conductance regulator (CFTR)* gene encodes a cAMP-regulated anion channel [1, 11]. CFTR is expressed in many tissues, but it is best known for its detrimental role in the development of cystic fibrosis*.* Mutations in the *CFTR* gene result in a lack of functional channels at the plasma membrane of epithelial cells and cause dysfunction of many affected organs such as the lungs, the intestine, and the pancreas [2, 12]. CFTR function, however, may also be impaired in inflammatory or infectious diseases. Rapid loss of functional CFTR has been observed following bacterial or viral infections, and among others, has been linked to pneumonia-induced acute respiratory distress syndrome (ARDS) [7]. ARDS is a syndrome of acute respiratory failure caused by noncardiogenic pulmonary edema. It is characterized by damage to the endothelial and epithelial barriers of the lung. The resulting increase in permeability to liquid and proteins across lung endothelial and epithelial barriers causes protein-rich edema in the lung interstitium and alveoli, respectively [9]. The mechanisms linking infection, loss of CFTR, and barrier dysfunction, however, have been elusive.

In an elegant study published in the *Science Translational Medicine*, Erfinanda and colleagues now identified an important pathomechanism that links infection to loss of functional CFTR from pulmonary endothelial cells, lung barrier failure, and development of pneumonia-induced ARDS [4]. First, they confirmed that CFTR expression is downregulated in human and murine lung tissue following infection with *S. pneumoniae* or *P. aeruginosa*. Of note, CFTR was also lost from endothelial cells treated with plasma from COVID patients (personal communication). In elaborate experiments, they further showed that loss of CFTR function increased endothelial permeability and edema formation in isolated perfused rat lungs. This is linked to an increase in intracellular Cl^−^ and Ca^2+^ levels within the endothelial cells. They then went on to fully delineate the molecular pathways that link loss of CFTR function to endothelial barrier failure. CFTR acts as an active ion channel in pulmonary endothelial cells and loss of CFTR reduces Cl^−^ conductance across the plasma membrane which results in an increase in the intracellular Cl^−^ concentration [Cl^−^]_i_. Counterintuitively, the authors found that inhibition of CFTR also leads to membrane depolarization, opening of voltage-gated calcium channels (VGCCs), and an increase in the intracellular Ca^2+^ concentration in endothelial cells. Since inhibition of a Cl^−^ conductance in a cell, where Cl^−^ is not passively distributed, will rather cause membrane hyperpolarization, the authors aimed at unraveling this conundrum. They found that the increase in [Cl^−^]_i_ inhibits the serine-threonine kinase with-no-lysine kinase 1 (WNK1). Inhibition of WNK1 causes endothelial Ca^2+^ influx via activation of the polymodal cation channel transient receptor potential vanilloid 4 (TRPV4), a known regulator of lung endothelial barrier function [10] (Fig. 1). TRPV4-deficient (*Trpv4*^*−/−*^) mice have reduced permeability-type lung edema upon *S*. *pneumoniae* infection, confirming TRPV4 as a downstream effector of endothelial barrier failure after CFTR and subsequent WNK1 inhibition.

**Fig. 1:**
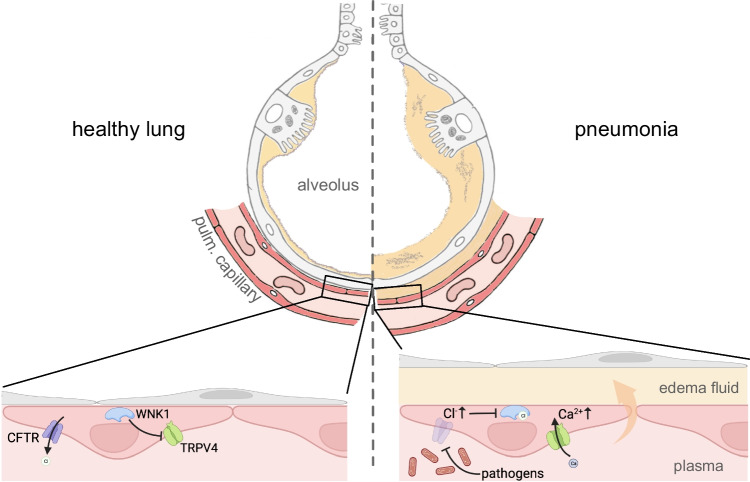
Permeability to liquid and proteins across the endothelial and epithelial barriers is tightly regulated in the healthy lung (left). This prevents formation of pulmonary edema and is critical for adequate gas exchange in the lung. Barrier-damage-induced pulmonary edema is a key feature of ARDS. Erfinanda and colleagues have now unraveled an important pathomechanism for lung barrier failure and development of pneumonia-induced ARDS. Infection causes a loss of CFTR from pulmonary endothelial cells resulting in an increase in intracellular Cl^−^ levels. High Cl^−^ levels inhibit WNK1, which in turn leads to activation (disinhibition) of TRPV4. This raises intracellular Ca^2+^ levels and leads to increased endothelial permeability and formation of pulmonary edema (right). Rescuing CFTR function with ivacaftor, a clinically approved CFTR potentiator, prevents barrier failure and edema

Finally, the translational potential of these findings was convincingly demonstrated in vitro and in vivo. Ivacaftor, a clinically approved CFTR potentiator [3], stabilizes endothelial CFTR expression and function, prevents endothelial barrier failure and edema, and improves survival in mice with *S. pneumoniae*–induced pneumonia. The relevance of these findings is supported by a very recent report, showing that SARS-CoV and SARS-CoV-2 cause a loss of CFTR expression and increased edema accumulation in lungs of mice which is rescued by ivacaftor [5].

Overall, the study of Erfinanda and colleagues presents a possible therapeutic strategy in people suffering from ARDS due to severe pneumonia. Such treatment is urgently needed. Despite five decades of basic and clinical research, there is still no effective pharmacotherapy for ARDS and the treatment remains primarily supportive [6]. Furthermore, these findings can expand the success story of the highly effective CFTR-directed therapeutics that are already a life-changing treatment for up to 90% of people with cystic fibrosis who carry responsive CFTR mutations [8].

## Data Availability

Not applicable.

## References

[1] Anderson MP, Gregory RJ, Thompson S, Souza DW, Paul S, Mulligan RC, Smith AE, Welsh MJ (1991). Demonstration that CFTR is a chloride channel by alteration of its anion selectivity. Science.

[2] Bombieri C, Seia M, Castellani C (2015). Genotypes and phenotypes in cystic fibrosis and cystic fibrosis transmembrane regulator-related disorders. Semin Respir Crit Care Med.

[3] Csanady L, Torocsik B (2019) Cystic fibrosis drug ivacaftor stimulates CFTR channels at picomolar concentrations. Elife 8:e46450. 10.7554/eLife.46450 10.7554/eLife.46450 PMC6594753 31205003

[4] Erfinanda L, Zou L, Gutbier B, Kneller L, Weidenfeld S, Michalick L, Lei D, Reppe K, Teixeira Alves LG, Schneider B, Zhang Q, Li C, Fatykhova D, Schneider P, Liedtke W, Sohara E, Mitchell TJ, Gruber AD, Hocke A, Hippenstiel S, Suttorp N, Olschewski A, Mall MA, Witzenrath M, Kuebler WM (2022) Loss of endothelial CFTR drives barrier failure and edema formation in lung infection and can be targeted by CFTR potentiation. Sci Transl Med 14:eabg8577. 10.1126/scitranslmed.abg8577 10.1126/scitranslmed.abg8577 36475904

[5] Honrubia JM, Gutierrez-Alvarez J, Sanz-Bravo A, Gonzalez-Miranda E, Munoz-Santos D, Castano-Rodriguez C, Wang L, Villarejo-Torres M, Ripoll-Gomez J, Esteban A, Fernandez-Delgado R, Sanchez-Cordon PJ, Oliveros JC, Perlman S, McCray PB Jr., Sola I, Enjuanes L (2023) SARS-CoV-2-mediated lung edema and replication are diminished by cystic fibrosis transmembrane conductance regulator modulators. mBio:e0313622. 10.1128/mbio.03136-22 10.1128/mbio.03136-22 PMC9973274 36625656

[6] Huppert LA, Matthay MA, Ware LB (2019). Pathogenesis of acute respiratory distress syndrome. Semin Respir Crit Care Med.

[7] Londino JD, Lazrak A, Collawn JF, Bebok Z, Harrod KS, Matalon S (2017). Influenza virus infection alters ion channel function of airway and alveolar cells: mechanisms and physiological sequelae. Am J Physiol Lung Cell Mol Physiol.

[8] Mall MA, Mayer-Hamblett N, Rowe SM (2020). Cystic fibrosis: emergence of highly effective targeted therapeutics and potential clinical implications. Am J Respir Crit Care Med.

[9] Matthay MA, Zemans RL (2011). The acute respiratory distress syndrome: pathogenesis and treatment. Annu Rev Pathol.

[10] Morty RE, Kuebler WM (2014). TRPV4: an exciting new target to promote alveolocapillary barrier function. Am J Physiol Lung Cell Mol Physiol.

[11] Rich DP, Gregory RJ, Anderson MP, Manavalan P, Smith AE, Welsh MJ (1991). Effect of deleting the R domain on CFTR-generated chloride channels. Science.

[12] Sosnay PR, Siklosi KR, Van Goor F, Kaniecki K, Yu H, Sharma N, Ramalho AS, Amaral MD, Dorfman R, Zielenski J, Masica DL, Karchin R, Millen L, Thomas PJ, Patrinos GP, Corey M, Lewis MH, Rommens JM, Castellani C, Penland CM, Cutting GR (2013). Defining the disease liability of variants in the cystic fibrosis transmembrane conductance regulator gene. Nat Genet.

